# Supplementary material for: Self-Assembled Bimetallic Aluminum-Salen Catalyst for the Cyclic Carbonates Synthesis
Source: Molecules. 2021 Jul 5;26(13):4097. doi: 10.3390/molecules26134097 (PMC8271522; doi:10.3390/molecules26134097)
Supplement: Supplementary file 1 [file molecules-26-04097-s001.zip › molecules-1235981-supplementary.pdf]

**Supporting Information**

# Self-Assembled Bimetallic Aluminum-Salen Catalyst for the Cyclic Carbonates Synthesis

**Wooyong Seong [1], Hyungwoo Hahm [1], Seyong Kim [1], Jongwoo Park [2,3], Khalil A. Abboud [2] and Sukwon Hong [1,4,*]**

[1] Department of Chemistry, Gwangju Institute of Science and Technology, Gwangju 61005, Korea; optimistwy@gist.ac.kr (W.S.); hyungwoo@gist.ac.kr (H.H.); timmy369@gist.ac.kr (S.K.)
[2] Department of Chemistry, University of Florida P.O.Box 117200, Gainesville, FL 32611-7200, USA; Jongwoo.Park@sk.com (J.P.); abboud@chem.ufl.edu (K.A.A.)
[3] SK biotek, 325 Exporo, Yuseong-gu, Daejeon 34124, Korea
[4] Gwangju Institute of Science and Technology, School of Materials Science and Engineering, Gwangju 61005, Korea
* Correspondence: shong@gist.ac.kr

**Table of contents**

| | |
|---|---|
| **1. Materials and instrumentation** | **SI-2** |
| **2. Catalyst preparation** | **SI-2** |
| **3. Cyclic carbonates synthesis** | **SI-3** |
| **4. X-ray experiment** | **SI-4** |
| **5. Crystal structure of bis-urea salen Ni complex (4, Park4)** | **SI-4** |
| **Table S1. Crystal data and structure refinement for park4.** | **SI-4** |
| **Table S2. Atomic coordinates ( x $10^4$) and equivalent isotropic displacement parameters (Å$^2$x $10^3$) for park4. U(eq) is defined as one third of the trace of the orthogonalized $U^{ij}$ tensor.** | **SI-7** |
| **Table S3. Bond lengths [Å] and angles [°] for park4.** | **SI-11** |
| **Table S4. Anisotropic displacement parameters (Å$^2$x $10^3$) for park4. The anisotropic displacement factor exponent takes the form: $-2\pi^2[ h^2 a^{*2}U^{11} + ... + 2 h k a^* b^* U^{12} ]$** | **SI-34** |
| **Table S5. Hydrogen coordinates ( x $10^4$) and isotropic displacement parameters (Å$^2$x $10^3$) for park4.** | **SI-37** |
| **Table S6. Hydrogen bonds for park4 [Å and °].** | **SI-40** |
| **6. Mass spectrum of a bis-urea salen aluminum catalyst (3b)** | **SI-41** |
| **7. FTIR spectrum of bis-urea salen aluminum catalyst (3b)** | **SI-44** |

## 1. Materials and instrumentation

Experiments involving moisture and/or air sensitive components were performed in flame-dried glassware under a positive pressure of argon (99.999% was purchased from Sinil gas (Co.)) and/or carbon dioxide (99.99% was purchased from Sinil gas (Co.), filtered over drying column that packed with activated carbon and 4 Å molecular sieve). Oxiranes (epoxides) were distilled by calcium hydride and stored over 4 Å molecular sieve. Other reagents were commercially purchased by sigma Aldrich (Co.) (Saint Louis, MO, USA), Acros Organics (Co.) (Somerset, N.J., USA) Alfa

Aeser (Co.) (Haverhill, MA, USA) and were used as received without further purification for the reaction. Tetrahydrofuran (THF), dichloromethan (DCM) and *N*-dimethylformamide (DMF) were dried by J.C. Meyer solvent purification system.

Proton nuclear magnetic resonance ($^1$H NMR) was performed on a JEOL 400 Mhz NMR spectrometers. Chemical shift $^1$H NMR spectra are reported as in units of ppm (part per million) downfield or up-field from $CDCl_3$ (7.26, singlet), DMSO-d6 (2.50, multiplet). High-resolution mass spectra (HRMS) were recorded on a JEOL JMS-700 MStation mass spectrometer. All FTIR samples were mixed with mineral oil as powder on KBr windows. IR spectra were obtained using an ATR-IR spectrometer (AlPHA ll) with a universal sampling module (A230/D, QuickSnap) as an accessory. The solution IR was obtained by using Speca (Co.) "Omni-Cell traditional liquid transmission cell" with "Omni-Cell PTFE spacers rectangular 0.10 mm" with KBr windows.

TLC was used on Merk (Co.) pre-coated 60 Å (F254) silica gel glass plates and the TLC spots were visualized under 254 nm, 365 nm UV light and iodine staining. All flash column chromatography was performed through Merk 230–400 Mesh 60 Å silica gel.

**2. Catalyst preparation**

Ligand 2a, 2b was prepared according to the literature[1].

Jacobson salen ligand aluminum chloride complex (**3a**) was prepared by following procedure. 437 mg (0.80 mmol, 1.00 equiv.) of Jacobson ligand (**2a**) and 0.84 mL of di(*iso*-butyl)aluminum chloride (DIBAL-Cl, *w/w* 25% in hexane, 0.80 mmol, 1 equiv.) placed in 50 mL Schlenk flask. 15 mL of dichloromethane (DCM) was added to flask. Stir for 20 hours in room temperature. After stir, the reaction mixture was dried under low pressure. And washed with 15 mL of dried hexane 3 times. 484 mg (99% yield) of yellow solid was prepared.

(R,R)-Bis(bis(3,5-trifluoromethylphenyl)ureido)-(salen)aluminum complex (**3b**) was prepared by following procedure. 91.6 mg (0.09 mmol, 1.00 equiv.) of (R,R)-Bis(bis(3,5-trifluoromethylphenyl)ureido)-(salen) (**2b**) and 0.10 mL of di(*iso*-butyl)aluminum chloride (DIBAL-Cl, *w/w* 25% in hexane, 0.09 mmol, 1.00 equiv.) placed in 10 mL Schlenk flask. 2 mL of dichloromethane (DCM) was added to flask. Stir for 20 hours in room temperature. After stir, the reaction mixture was dried under low pressure. And the crude solid was washed with 3 mL of dried hexane, 3 times. 110 mg (99% yield) of white-yellow solid was prepared.

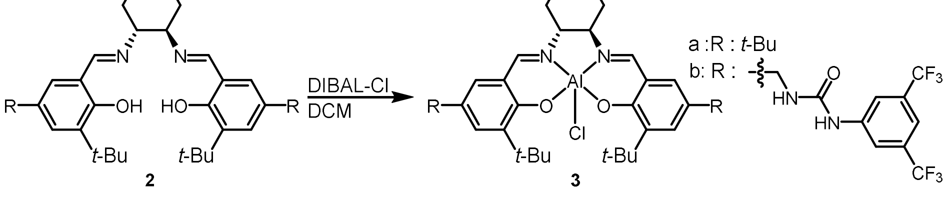


1. Park, J.; Lang, K.; Abboud, K. A.; Hong, S.; *Chem. Eur. J.* **2011**, *17*, 2236–2245.

**3. Cyclic carbonates synthesis**

1 bar of $CO_2$ and 45 °C reaction condition

5000 equiv. of dried oxirane (47.0 mmol) was placed in a Teflon capped Schlenk flask, 1 equiv. (0.009 mmol) of catalyst and 1 equiv. (0.009 mmol) of ammonium halide were added to the flask. The mixture was degassed by lyophilization 3 times. The degassed mixture was heated up to room temperature. Dried $CO_2$ was packed in the flask and Teflon cap was closed. The mixture in flask was stirred for 3 hours at 45 °C. After stirred, the reaction mixture was cooled down to room temperature and filtered over silica-gel (5 cm) with DCM. The filtrate was concentrated under reduced pressure. Clear or off yellow oil was prepared.

10 bar of $CO_2$ and 90 ˚C reaction condition

5000 equiv. of dried oxirane (47.0 mmol) was placed in in a 10 mL stainless steel bomb reactor, 1 equiv. (0.009 mmol) of catalyst and 1 equiv. (0.009 mmol) of ammonium halide were added to the reactor. The reactor was purged to 5 bar of dried $CO_2$, 3 times. 10 bar of dried $CO_2$ was packed in the reactor through sealed gas connector. The reactor was stirred for 3 hours at 90 °C. After stirred the reaction mixture was cooled down to ambient temperature (ice bath, 30 min.) and filtered over silica-gel (5 cm) with DCM. The filtrate was concentrated under reduced pressure. Clear or off yellow oil was prepared.

**4. X-ray experiment**

Data were collected at 173 K on a Siemens SMART PLATFORM equipped with A CCD area detector and a graphite monochromator utilizing MoK$_\alpha$ radiation ($\lambda$ = 0.71073 Å). Cell parameters were refined using up to 8192 reflections. A full sphere of data (1850 frames) was collected using the ω-scan method (0.3˚ frame width). The first 50 frames were re-measured at the end of data collection to monitor instrument and crystal stability (maximum correction on I was < 1 %). Absorption corrections by integration were applied based on measured indexed crystal faces.

The structure was solved by the Direct Methods in *SHELXTL6*, and refined using full-matrix least squares. The non-H atoms were treated anisotropically, whereas the hydrogen atoms were calculated in ideal positions and were riding on their respective carbon atoms. The asymmetric unit consists of the Ni complex and a dimethylfromamide solvent molecule. All four CF3 groups have the fluorine atoms disordered. Each group was refined in two parts with their site occupation factors fixed at 0.5 after having refined to near this value. The C atoms, with their protons, are also disordered. They are atoms C1, C4 and C6. They were also refined in two parts each and given 0.5 values for each

disordered part. A total of 686 parameters were refined in the final cycle of refinement using 9198 reflections with I > 2σ(I) to yield $R_1$ and $wR_2$ of 5.51% and 11.03%, respectively. Refinement was done using $F^2$.

*SHELXTL6* (2000). Bruker-AXS, Madison, Wisconsin, USA.

**5. Crystal structure of bis-urea salen Ni complex (4)**

CCDC : 2080102, 2080103

**Table S1.** Crystal data and structure refinement for park4.

| | | |
|---|---|---|
| Identification code | park4 | |
| Empirical formula | C51 H55 F12 N7 Ni O5 | |
| Formula weight | 1132.73 | |
| Temperature | 100(2) K | |
| Wavelength | 0.71073 Å | |
| Crystal system | Triclinic | |
| Space group | P-1 | |
| Unit cell dimensions | a = 9.3525(13) Å | α = 91.018(3)°. |
| | b = 13.0965(17) Å | β = 97.294(2)°. |
| | c = 20.515(3) Å | γ = 94.674(3)°. |
| Volume | 2483.2(6) Å $^3$ | |
| Z | 2 | |
| Density (calculated) | 1.515 Mg/m$^3$ | |
| Absorption coefficient | 0.492 mm$^{-1}$ | |
| F(000) | 1172 | |
| Crystal size | 0.31 × 0.12 × 0.05 mm$^3$ | |
| Theta range for data collection | 1.56 to 27.50°. | |
| Index ranges | −12 ≤ h ≤ 12, −14 ≤ k ≤ 17, −26 ≤ l ≤ 24 | |
| Reflections collected | 27913 | |

| | |
|---|---|
| Independent reflections | 11151 [R(int) = 0.0294] |
| Completeness to theta = 27.50° | 97.9 % |
| Absorption correction | None |
| Max. and min. transmission | 0.9768 and 0.8632 |
| Refinement method | Full-matrix least-squares on $F^2$ |
| Data / restraints / parameters | 11151 / 0 / 686 |
| Goodness-of-fit on $F^2$ | 1.158 |
| Final R indices [I>2sigma(I)] | R1 = 0.0551, wR2 = 0.1103 [9198] |
| R indices (all data) | R1 = 0.0695, wR2 = 0.1151 |
| Largest diff. peak and hole | 0.585 and -0.615 e.Å$^{-3}$ |

$R1 = \Sigma(||F_o| - |F_c||) / \Sigma|F_o|$ $wR2 = [\Sigma[w(F_o^2 - F_c^2)^2] / \Sigma[w(F_o^2)^2]]^{1/2}$

$S = [\Sigma[w(F_o^2 - F_c^2)^2] / (n-p)]^{1/2}$ $w= 1/[\sigma^2(F_o^2)+(m*p)^2+n*p]$, $p = [max(F_o^2,0)+ 2* F_c^2]/3$, m & n are constants.

**Table S2.** Atomic coordinates ( × $10^4$) and equivalent isotropic displacement parameters (Å$^2$ × $10^3$) for park4. U(eq) is defined as one third of the trace of the orthogonalized $U^{ij}$ tensor.

| | x | y | z | U(eq) |
|---|---|---|---|---|
| Ni1 | 2500(1) | -777(1) | -137(1) | 12(1) |
| F1 | 12614(5) | 4737(4) | 3837(2) | 24(1) |
| F2 | 12138(4) | 4227(3) | 4785(2) | 18(1) |
| F3 | 11569(5) | 5736(4) | 4381(2) | 18(1) |
| F1' | 12473(5) | 4941(4) | 3704(2) | 26(1) |
| F2' | 12376(5) | 4196(4) | 4624(2) | 26(1) |
| F3' | 11496(5) | 5639(4) | 4542(2) | 20(1) |
| F4 | 5311(5) | 4424(3) | 3058(2) | 18(1) |
| F5 | 6520(5) | 5782(3) | 3497(2) | 20(1) |
| F6 | 5742(5) | 4707(4) | 4183(3) | 22(1) |
| F4' | 5161(5) | 4296(3) | 3204(2) | 18(1) |
| F5' | 6573(5) | 5744(4) | 3305(2) | 24(1) |
| F6' | 5816(6) | 4927(4) | 4109(3) | 27(2) |
| F7 | 62(6) | 1141(5) | -5316(3) | 25(2) |
| F8 | 747(5) | 1271(4) | -4273(2) | 20(1) |
| F9 | 50(6) | 2573(4) | -4849(3) | 24(1) |
| F7' | -130(6) | 1238(5) | -5415(3) | 25(2) |
| F8' | 716(5) | 1054(4) | -4396(2) | 23(1) |
| F9' | 231(6) | 2570(4) | -4704(3) | 23(1) |
| F10 | -6715(6) | 1967(4) | -5050(3) | 27(2) |
| F11 | -5334(7) | 2648(5) | -5679(3) | 23(2) |
| F12 | -5169(5) | 3281(3) | -4779(2) | 20(1) |

| | | | | |
|---|---|---|---|---|
| F10' | -6737(5) | 1798(4) | -5169(3) | 21(1) |
| F11' | -5156(7) | 2578(5) | -5746(3) | 25(2) |
| F12' | -5502(5) | 3122(4) | -4690(2) | 25(1) |
| O1 | 3395(2) | -1684(2) | 432(1) | 18(1) |
| O2 | 1919(2) | -1909(2) | -690(1) | 17(1) |
| O3 | 6598(2) | 1055(2) | 3208(1) | 18(1) |
| O4 | -1802(2) | -1189(2) | -3436(1) | 17(1) |
| O5 | 617(3) | 8947(2) | 2416(1) | 31(1) |
| N1 | 3242(3) | 379(2) | 360(1) | 21(1) |
| N2 | 1422(3) | 94(2) | -651(1) | 21(1) |
| N3 | 8210(3) | 62(2) | 2831(1) | 17(1) |
| N4 | 8917(3) | 1729(2) | 3148(1) | 16(1) |
| N5 | -3669(3) | -1632(2) | -2861(1) | 15(1) |
| N6 | -3889(3) | -365(2) | -3613(1) | 17(1) |
| N7 | 1690(3) | 7604(2) | 2895(1) | 24(1) |
| C1 | 2908(7) | 1360(4) | 39(3) | 14(1) |
| C6 | 1366(6) | 1111(4) | -279(3) | 15(1) |
| C1' | 2332(7) | 1277(5) | 195(3) | 16(1) |
| C6' | 1976(6) | 1195(4) | -546(3) | 12(1) |
| C2 | 3037(3) | 2306(2) | 460(2) | 22(1) |
| C3 | 2179(4) | 3163(3) | 175(2) | 27(1) |
| C4 | 845(7) | 2925(5) | -219(3) | 21(1) |
| C4' | 1529(6) | 3044(5) | -563(3) | 18(1) |
| C5 | 870(3) | 1975(2) | -734(2) | 23(1) |
| C7 | 4215(4) | 408(2) | 867(2) | 23(1) |
| C8 | 4757(3) | -462(2) | 1200(1) | 17(1) |
| C9 | 4239(3) | -1479(2) | 987(1) | 14(1) |

| | | | | |
|---|---|---|---|---|
| C10 | 4684(3) | -2303(2) | 1402(1) | 15(1) |
| C11 | 5664(3) | -2059(2) | 1958(1) | 15(1) |
| C12 | 6236(3) | -1052(2) | 2148(1) | 16(1) |
| C13 | 5758(3) | -264(2) | 1770(1) | 16(1) |
| C14 | 4039(3) | -3413(2) | 1241(1) | 16(1) |
| C15 | 2376(3) | -3460(2) | 1210(2) | 19(1) |
| C16 | 4437(3) | -3802(2) | 586(2) | 24(1) |
| C17 | 4590(3) | -4160(2) | 1768(2) | 22(1) |
| C18 | 421(3) | -164(2) | -1129(2) | 20(1) |
| C19 | 47(3) | -1181(2) | -1392(1) | 14(1) |
| C20 | 870(3) | -2003(2) | -1179(1) | 14(1) |
| C21 | 520(3) | -2978(2) | -1523(1) | 13(1) |
| C22 | -727(3) | -3092(2) | -1968(1) | 15(1) |
| C23 | -1623(3) | -2296(2) | -2132(1) | 14(1) |
| C24 | -1204(3) | -1342(2) | -1861(1) | 14(1) |
| C25 | 1494(3) | -3855(2) | -1384(2) | 17(1) |
| C26 | 3068(3) | -3478(3) | -1472(2) | 26(1) |
| C27 | 1430(4) | -4226(2) | -682(2) | 25(1) |
| C28 | 1038(3) | -4778(2) | -1863(2) | 20(1) |
| C29 | 7251(3) | -879(2) | 2787(1) | 18(1) |
| C30 | 7835(3) | 951(2) | 3071(1) | 14(1) |
| C31 | 8848(3) | 2716(2) | 3404(1) | 14(1) |
| C32 | 10149(3) | 3232(2) | 3691(1) | 15(1) |
| C33 | 10184(3) | 4230(2) | 3927(1) | 16(1) |
| C34 | 8941(3) | 4748(2) | 3899(1) | 17(1) |
| C35 | 7651(3) | 4224(2) | 3612(1) | 16(1) |
| C36 | 7579(3) | 3222(2) | 3370(1) | 15(1) |

| | | | | |
|---|---|---|---|---|
| C37 | 11626(3) | 4745(2) | 4221(2) | 21(1) |
| C38 | 6304(3) | 4787(2) | 3575(2) | 22(1) |
| C39 | -3019(3) | -2535(2) | -2592(1) | 16(1) |
| C40 | -3030(3) | -1076(2) | -3312(1) | 14(1) |
| C41 | -3491(3) | 356(2) | -4066(1) | 15(1) |
| C42 | -2062(3) | 578(2) | -4196(1) | 16(1) |
| C43 | -1764(3) | 1333(2) | -4633(2) | 18(1) |
| C44 | -2835(3) | 1890(2) | -4953(1) | 19(1) |
| C45 | -4240(3) | 1668(2) | -4819(1) | 18(1) |
| C46 | -4581(3) | 911(2) | -4384(1) | 17(1) |
| C47 | -226(3) | 1560(2) | -4775(2) | 21(1) |
| C48 | -5389(3) | 2336(2) | -5096(2) | 22(1) |
| C49 | 1689(4) | 8510(3) | 2613(2) | 24(1) |
| C50 | 324(4) | 7038(3) | 2988(2) | 36(1) |
| C51 | 3004(4) | 7180(3) | 3178(2) | 34(1) |

**Table S3.** Bond lengths [Å] and angles [°] for park4.

| | |
|---|---|
| Ni1-N2 | 1.842(2) |
| Ni1-N1 | 1.846(3) |
| Ni1-O2 | 1.853(2) |
| Ni1-O1 | 1.855(2) |
| F1-C37 | 1.289(5) |
| F2-C37 | 1.403(5) |
| F3-C37 | 1.340(5) |
| F1'-C37 | 1.417(6) |
| F2'-C37 | 1.284(5) |
| F3'-C37 | 1.355(6) |
| F4-C38 | 1.368(5) |
| F5-C38 | 1.318(5) |
| F6-C38 | 1.417(6) |
| F4'-C38 | 1.341(5) |
| F5'-C38 | 1.397(6) |
| F6'-C38 | 1.256(6) |
| F7-C47 | 1.297(7) |
| F8-C47 | 1.365(5) |
| F9-C47 | 1.346(6) |
| F7'-C47 | 1.389(7) |
| F8'-C47 | 1.327(6) |
| F9'-C47 | 1.355(6) |
| F10-C48 | 1.309(6) |
| F11-C48 | 1.276(7) |
| F12-C48 | 1.375(5) |

| | |
|---|---|
| F10'-C48 | 1.382(6) |
| F11'-C48 | 1.415(7) |
| F12'-C48 | 1.332(6) |
| O1-C9 | 1.310(3) |
| O2-C20 | 1.307(3) |
| O3-C30 | 1.242(3) |
| O4-C40 | 1.227(3) |
| O5-C49 | 1.223(4) |
| N1-C7 | 1.290(4) |
| N1-C1 | 1.493(6) |
| N1-C1' | 1.525(6) |
| N2-C18 | 1.287(4) |
| N2-C6' | 1.495(6) |
| N2-C6 | 1.531(6) |
| N3-C30 | 1.346(4) |
| N3-C29 | 1.458(4) |
| N3-H3 | 0.79(4) |
| N4-C30 | 1.369(4) |
| N4-C31 | 1.395(4) |
| N4-H4 | 0.82(3) |
| N5-C40 | 1.355(4) |
| N5-C39 | 1.458(4) |
| N5-H5 | 0.89(3) |
| N6-C40 | 1.383(4) |
| N6-C41 | 1.399(4) |
| N6-H6 | 0.86(4) |
| N7-C49 | 1.330(4) |

| | |
|---|---|
| N7-C51 | 1.448(4) |
| N7-C50 | 1.460(5) |
| C1-C2 | 1.486(6) |
| C1-C6 | 1.513(8) |
| C1-H1A | 1.0000 |
| C6-C5 | 1.543(6) |
| C6-H6A | 1.0000 |
| C1'-C2 | 1.512(7) |
| C1'-C6' | 1.514(8) |
| C1'-H1'A | 1.0000 |
| C6'-C5 | 1.532(6) |
| C6'-H6'A | 1.0000 |
| C2-C3 | 1.515(4) |
| C2-H2A | 0.9900 |
| C2-H2B | 0.9900 |
| C2-H2C | 0.9900 |
| C2-H2D | 0.9900 |
| C3-C4 | 1.407(7) |
| C3-C4' | 1.559(7) |
| C3-H3A | 0.9900 |
| C3-H3B | 0.9900 |
| C3-H3C | 0.9900 |
| C3-H3D | 0.9900 |
| C4-C5 | 1.622(7) |
| C4-H4A | 0.9900 |
| C4-H4B | 0.9900 |
| C4'-C5 | 1.499(7) |

| | |
|---|---|
| C4'-H4'A | 0.9900 |
| C4'-H4'B | 0.9900 |
| C5-H5A | 0.9900 |
| C5-H5B | 0.9900 |
| C5-H5C | 0.9900 |
| C5-H5D | 0.9900 |
| C7-C8 | 1.433(4) |
| C7-H7A | 0.9500 |
| C8-C13 | 1.410(4) |
| C8-C9 | 1.421(4) |
| C9-C10 | 1.442(4) |
| C10-C11 | 1.384(4) |
| C10-C14 | 1.542(4) |
| C11-C12 | 1.412(4) |
| C11-H11A | 0.9500 |
| C12-C13 | 1.371(4) |
| C12-C29 | 1.519(4) |
| C13-H13A | 0.9500 |
| C14-C16 | 1.530(4) |
| C14-C17 | 1.540(4) |
| C14-C15 | 1.545(4) |
| C15-H15A | 0.9800 |
| C15-H15B | 0.9800 |
| C15-H15C | 0.9800 |
| C16-H16A | 0.9800 |
| C16-H16B | 0.9800 |
| C16-H16C | 0.9800 |

| | |
|---|---|
| C17-H17A | 0.9800 |
| C17-H17B | 0.9800 |
| C17-H17C | 0.9800 |
| C18-C19 | 1.428(4) |
| C18-H18A | 0.9500 |
| C19-C24 | 1.416(4) |
| C19-C20 | 1.417(4) |
| C20-C21 | 1.441(4) |
| C21-C22 | 1.383(4) |
| C21-C25 | 1.532(4) |
| C22-C23 | 1.409(4) |
| C22-H22A | 0.9500 |
| C23-C24 | 1.365(4) |
| C23-C39 | 1.518(4) |
| C24-H24A | 0.9500 |
| C25-C27 | 1.535(4) |
| C25-C28 | 1.541(4) |
| C25-C26 | 1.548(4) |
| C26-H26A | 0.9800 |
| C26-H26B | 0.9800 |
| C26-H26C | 0.9800 |
| C27-H27A | 0.9800 |
| C27-H27B | 0.9800 |
| C27-H27C | 0.9800 |
| C28-H28A | 0.9800 |
| C28-H28B | 0.9800 |
| C28-H28C | 0.9800 |

| | |
|---|---|
| C29-H29A | 0.9900 |
| C29-H29B | 0.9900 |
| C31-C32 | 1.397(4) |
| C31-C36 | 1.401(4) |
| C32-C33 | 1.383(4) |
| C32-H32A | 0.9500 |
| C33-C34 | 1.388(4) |
| C33-C37 | 1.506(4) |
| C34-C35 | 1.394(4) |
| C34-H34A | 0.9500 |
| C35-C36 | 1.387(4) |
| C35-C38 | 1.505(4) |
| C36-H36A | 0.9500 |
| C39-H39A | 0.9900 |
| C39-H39B | 0.9900 |
| C41-C46 | 1.401(4) |
| C41-C42 | 1.404(4) |
| C42-C43 | 1.382(4) |
| C42-H42A | 0.9500 |
| C43-C44 | 1.392(4) |
| C43-C47 | 1.510(4) |
| C44-C45 | 1.385(4) |
| C44-H44A | 0.9500 |
| C45-C46 | 1.390(4) |
| C45-C48 | 1.503(4) |
| C46-H46A | 0.9500 |
| C49-H49A | 0.9500 |

| | |
|---|---|
| C50-H50A | 0.9800 |
| C50-H50B | 0.9800 |
| C50-H50C | 0.9800 |
| C51-H51A | 0.9800 |
| C51-H51B | 0.9800 |
| C51-H51C | 0.9800 |
| | |
| N2-Ni1-N1 | 85.93(11) |
| N2-Ni1-O2 | 94.02(10) |
| N1-Ni1-O2 | 173.75(11) |
| N2-Ni1-O1 | 173.20(11) |
| N1-Ni1-O1 | 94.74(10) |
| O2-Ni1-O1 | 86.05(9) |
| C9-O1-Ni1 | 128.39(19) |
| C20-O2-Ni1 | 128.00(18) |
| C7-N1-C1 | 118.6(3) |
| C7-N1-C1' | 121.8(3) |
| C1-N1-C1' | 25.4(3) |
| C7-N1-Ni1 | 125.9(2) |
| C1-N1-Ni1 | 113.9(3) |
| C1'-N1-Ni1 | 111.0(3) |
| C18-N2-C6' | 119.4(3) |
| C18-N2-C6 | 118.4(3) |
| C6'-N2-C6 | 32.3(3) |
| C18-N2-Ni1 | 126.7(2) |
| C6'-N2-Ni1 | 113.2(2) |
| C6-N2-Ni1 | 110.3(2) |

| | |
|---|---|
| C30-N3-C29 | 122.3(3) |
| C30-N3-H3 | 122(3) |
| C29-N3-H3 | 115(3) |
| C30-N4-C31 | 127.2(2) |
| C30-N4-H4 | 115(2) |
| C31-N4-H4 | 116(2) |
| C40-N5-C39 | 119.8(2) |
| C40-N5-H5 | 116(2) |
| C39-N5-H5 | 121(2) |
| C40-N6-C41 | 126.4(2) |
| C40-N6-H6 | 119(2) |
| C41-N6-H6 | 114(2) |
| C49-N7-C51 | 122.7(3) |
| C49-N7-C50 | 120.0(3) |
| C51-N7-C50 | 117.0(3) |
| C2-C1-N1 | 118.1(4) |
| C2-C1-C6 | 111.0(4) |
| N1-C1-C6 | 102.0(4) |
| C2-C1-H1A | 108.5 |
| N1-C1-H1A | 108.5 |
| C6-C1-H1A | 108.5 |
| C1-C6-N2 | 103.7(4) |
| C1-C6-C5 | 110.8(4) |
| N2-C6-C5 | 112.9(4) |
| C1-C6-H6A | 109.8 |
| N2-C6-H6A | 109.8 |
| C5-C6-H6A | 109.8 |

| | |
|---|---|
| C2-C1'-C6' | 114.8(5) |
| C2-C1'-N1 | 114.5(4) |
| C6'-C1'-N1 | 103.8(4) |
| C2-C1'-H1'A | 107.8 |
| C6'-C1'-H1'A | 107.8 |
| N1-C1'-H1'A | 107.8 |
| N2-C6'-C1' | 101.8(4) |
| N2-C6'-C5 | 115.7(4) |
| C1'-C6'-C5 | 106.4(4) |
| N2-C6'-H6'A | 110.8 |
| C1'-C6'-H6'A | 110.8 |
| C5-C6'-H6'A | 110.8 |
| C1-C2-C1' | 25.6(3) |
| C1-C2-C3 | 115.0(3) |
| C1'-C2-C3 | 110.4(3) |
| C1-C2-H2A | 108.5 |
| C1'-C2-H2A | 130.6 |
| C3-C2-H2A | 108.5 |
| C1-C2-H2B | 108.5 |
| C1'-C2-H2B | 87.5 |
| C3-C2-H2B | 108.5 |
| H2A-C2-H2B | 107.5 |
| C1-C2-H2C | 84.8 |
| C1'-C2-H2C | 109.6 |
| C3-C2-H2C | 109.6 |
| H2A-C2-H2C | 26.7 |
| H2B-C2-H2C | 128.6 |

| | |
|---|---|
| C1-C2-H2D | 125.7 |
| C1'-C2-H2D | 109.6 |
| C3-C2-H2D | 109.6 |
| H2A-C2-H2D | 84.0 |
| H2B-C2-H2D | 25.0 |
| H2C-C2-H2D | 108.1 |
| C4-C3-C2 | 119.8(4) |
| C4-C3-C4' | 39.7(3) |
| C2-C3-C4' | 117.0(3) |
| C4-C3-H3A | 107.4 |
| C2-C3-H3A | 107.4 |
| C4'-C3-H3A | 134.1 |
| C4-C3-H3B | 107.4 |
| C2-C3-H3B | 107.4 |
| C4'-C3-H3B | 71.2 |
| H3A-C3-H3B | 106.9 |
| C4-C3-H3C | 130.7 |
| C2-C3-H3C | 108.0 |
| C4'-C3-H3C | 108.0 |
| H3A-C3-H3C | 66.2 |
| H3B-C3-H3C | 42.6 |
| C4-C3-H3D | 70.0 |
| C2-C3-H3D | 108.0 |
| C4'-C3-H3D | 108.0 |
| H3A-C3-H3D | 43.6 |
| H3B-C3-H3D | 139.7 |
| H3C-C3-H3D | 107.3 |

| | |
|---|---|
| C3-C4-C5 | 113.1(4) |
| C3-C4-H4A | 108.9 |
| C5-C4-H4A | 108.9 |
| C3-C4-H4B | 108.9 |
| C5-C4-H4B | 108.9 |
| H4A-C4-H4B | 107.8 |
| C5-C4'-C3 | 111.7(4) |
| C5-C4'-H4'A | 109.3 |
| C3-C4'-H4'A | 109.3 |
| C5-C4'-H4'B | 109.3 |
| C3-C4'-H4'B | 109.3 |
| H4'A-C4'-H4'B | 108.0 |
| C4'-C5-C6' | 110.5(4) |
| C4'-C5-C6 | 117.7(4) |
| C6'-C5-C6 | 31.7(3) |
| C4'-C5-C4 | 37.8(3) |
| C6'-C5-C4 | 116.8(4) |
| C6-C5-C4 | 102.5(4) |
| C4'-C5-H5A | 125.8 |
| C6'-C5-H5A | 123.4 |
| C6-C5-H5A | 111.3 |
| C4-C5-H5A | 111.3 |
| C4'-C5-H5B | 73.5 |
| C6'-C5-H5B | 79.5 |
| C6-C5-H5B | 111.3 |
| C4-C5-H5B | 111.3 |
| H5A-C5-H5B | 109.2 |

| | |
|---|---|
| C4'-C5-H5C | 109.6 |
| C6'-C5-H5C | 109.6 |
| C6-C5-H5C | 126.8 |
| C4-C5-H5C | 130.3 |
| H5A-C5-H5C | 49.1 |
| H5B-C5-H5C | 60.2 |
| C4'-C5-H5D | 109.6 |
| C6'-C5-H5D | 109.6 |
| C6-C5-H5D | 78.3 |
| C4-C5-H5D | 72.7 |
| H5A-C5-H5D | 59.1 |
| H5B-C5-H5D | 167.7 |
| H5C-C5-H5D | 108.1 |
| N1-C7-C8 | 126.0(3) |
| N1-C7-H7A | 117.0 |
| C8-C7-H7A | 117.0 |
| C13-C8-C9 | 121.4(3) |
| C13-C8-C7 | 117.1(3) |
| C9-C8-C7 | 121.4(3) |
| O1-C9-C8 | 122.4(3) |
| O1-C9-C10 | 119.8(3) |
| C8-C9-C10 | 117.7(2) |
| C11-C10-C9 | 118.0(3) |
| C11-C10-C14 | 121.4(3) |
| C9-C10-C14 | 120.6(2) |
| C10-C11-C12 | 124.0(3) |
| C10-C11-H11A | 118.0 |

| | |
|---|---|
| C12-C11-H11A | 118.0 |
| C13-C12-C11 | 118.0(3) |
| C13-C12-C29 | 122.9(3) |
| C11-C12-C29 | 119.0(3) |
| C12-C13-C8 | 120.7(3) |
| C12-C13-H13A | 119.6 |
| C8-C13-H13A | 119.6 |
| C16-C14-C17 | 107.0(2) |
| C16-C14-C10 | 111.2(2) |
| C17-C14-C10 | 112.1(2) |
| C16-C14-C15 | 109.5(2) |
| C17-C14-C15 | 107.5(2) |
| C10-C14-C15 | 109.3(2) |
| C14-C15-H15A | 109.5 |
| C14-C15-H15B | 109.5 |
| H15A-C15-H15B | 109.5 |
| C14-C15-H15C | 109.5 |
| H15A-C15-H15C | 109.5 |
| H15B-C15-H15C | 109.5 |
| C14-C16-H16A | 109.5 |
| C14-C16-H16B | 109.5 |
| H16A-C16-H16B | 109.5 |
| C14-C16-H16C | 109.5 |
| H16A-C16-H16C | 109.5 |
| H16B-C16-H16C | 109.5 |
| C14-C17-H17A | 109.5 |
| C14-C17-H17B | 109.5 |

| | |
|---|---|
| H17A-C17-H17B | 109.5 |
| C14-C17-H17C | 109.5 |
| H17A-C17-H17C | 109.5 |
| H17B-C17-H17C | 109.5 |
| N2-C18-C19 | 125.2(3) |
| N2-C18-H18A | 117.4 |
| C19-C18-H18A | 117.4 |
| C24-C19-C20 | 121.3(3) |
| C24-C19-C18 | 117.3(3) |
| C20-C19-C18 | 121.4(3) |
| O2-C20-C19 | 122.8(3) |
| O2-C20-C21 | 119.5(2) |
| C19-C20-C21 | 117.7(2) |
| C22-C21-C20 | 117.7(3) |
| C22-C21-C25 | 121.9(3) |
| C20-C21-C25 | 120.4(2) |
| C21-C22-C23 | 123.9(3) |
| C21-C22-H22A | 118.1 |
| C23-C22-H22A | 118.1 |
| C24-C23-C22 | 118.3(3) |
| C24-C23-C39 | 122.8(3) |
| C22-C23-C39 | 118.9(3) |
| C23-C24-C19 | 120.4(3) |
| C23-C24-H24A | 119.8 |
| C19-C24-H24A | 119.8 |
| C21-C25-C27 | 110.2(2) |
| C21-C25-C28 | 112.0(2) |

| | |
|---|---|
| C27-C25-C28 | 108.0(2) |
| C21-C25-C26 | 109.3(2) |
| C27-C25-C26 | 110.0(3) |
| C28-C25-C26 | 107.3(2) |
| C25-C26-H26A | 109.5 |
| C25-C26-H26B | 109.5 |
| H26A-C26-H26B | 109.5 |
| C25-C26-H26C | 109.5 |
| H26A-C26-H26C | 109.5 |
| H26B-C26-H26C | 109.5 |
| C25-C27-H27A | 109.5 |
| C25-C27-H27B | 109.5 |
| H27A-C27-H27B | 109.5 |
| C25-C27-H27C | 109.5 |
| H27A-C27-H27C | 109.5 |
| H27B-C27-H27C | 109.5 |
| C25-C28-H28A | 109.5 |
| C25-C28-H28B | 109.5 |
| H28A-C28-H28B | 109.5 |
| C25-C28-H28C | 109.5 |
| H28A-C28-H28C | 109.5 |
| H28B-C28-H28C | 109.5 |
| N3-C29-C12 | 115.3(2) |
| N3-C29-H29A | 108.4 |
| C12-C29-H29A | 108.4 |
| N3-C29-H29B | 108.4 |
| C12-C29-H29B | 108.4 |

| | |
|---|---|
| H29A-C29-H29B | 107.5 |
| O3-C30-N3 | 122.4(3) |
| O3-C30-N4 | 122.8(3) |
| N3-C30-N4 | 114.9(2) |
| N4-C31-C32 | 116.9(2) |
| N4-C31-C36 | 124.3(3) |
| C32-C31-C36 | 118.8(3) |
| C33-C32-C31 | 120.5(3) |
| C33-C32-H32A | 119.7 |
| C31-C32-H32A | 119.7 |
| C32-C33-C34 | 121.7(3) |
| C32-C33-C37 | 117.7(3) |
| C34-C33-C37 | 120.6(3) |
| C33-C34-C35 | 117.2(3) |
| C33-C34-H34A | 121.4 |
| C35-C34-H34A | 121.4 |
| C36-C35-C34 | 122.5(3) |
| C36-C35-C38 | 120.2(3) |
| C34-C35-C38 | 117.3(3) |
| C35-C36-C31 | 119.3(3) |
| C35-C36-H36A | 120.4 |
| C31-C36-H36A | 120.4 |
| F2'-C37-F1 | 89.2(4) |
| F2'-C37-F3 | 118.3(4) |
| F1-C37-F3 | 105.0(4) |
| F2'-C37-F3' | 107.0(4) |
| F1-C37-F3' | 117.5(4) |

| | |
|---|---|
| F3-C37-F3' | 15.8(3) |
| F2'-C37-F2 | 17.4(3) |
| F1-C37-F2 | 106.3(4) |
| F3-C37-F2 | 109.1(3) |
| F3'-C37-F2 | 95.4(3) |
| F2'-C37-F1' | 105.3(4) |
| F1-C37-F1' | 16.2(3) |
| F3-C37-F1' | 94.8(4) |
| F3'-C37-F1' | 109.2(4) |
| F2-C37-F1' | 122.1(3) |
| F2'-C37-C33 | 114.5(3) |
| F1-C37-C33 | 114.0(3) |
| F3-C37-C33 | 113.1(3) |
| F3'-C37-C33 | 112.4(3) |
| F2-C37-C33 | 109.1(3) |
| F1'-C37-C33 | 108.1(3) |
| F6'-C38-F5 | 91.6(4) |
| F6'-C38-F4' | 102.1(4) |
| F5-C38-F4' | 116.7(4) |
| F6'-C38-F4 | 115.8(4) |
| F5-C38-F4 | 106.0(3) |
| F4'-C38-F4 | 16.2(3) |
| F6'-C38-F5' | 107.9(4) |
| F5-C38-F5' | 16.9(3) |
| F4'-C38-F5' | 107.7(3) |
| F4-C38-F5' | 94.5(3) |
| F6'-C38-F6 | 12.6(4) |

| | |
|---|---|
| F5-C38-F6 | 104.2(4) |
| F4'-C38-F6 | 96.8(4) |
| F4-C38-F6 | 112.1(4) |
| F5'-C38-F6 | 120.5(4) |
| F6'-C38-C35 | 116.1(3) |
| F5-C38-C35 | 114.4(3) |
| F4'-C38-C35 | 113.5(3) |
| F4-C38-C35 | 111.1(3) |
| F5'-C38-C35 | 109.0(3) |
| F6-C38-C35 | 108.9(3) |
| N5-C39-C23 | 114.2(2) |
| N5-C39-H39A | 108.7 |
| C23-C39-H39A | 108.7 |
| N5-C39-H39B | 108.7 |
| C23-C39-H39B | 108.7 |
| H39A-C39-H39B | 107.6 |
| O4-C40-N5 | 123.1(3) |
| O4-C40-N6 | 123.4(3) |
| N5-C40-N6 | 113.5(2) |
| N6-C41-C46 | 117.4(3) |
| N6-C41-C42 | 123.6(3) |
| C46-C41-C42 | 118.8(3) |
| C43-C42-C41 | 119.5(3) |
| C43-C42-H42A | 120.2 |
| C41-C42-H42A | 120.2 |
| C42-C43-C44 | 122.3(3) |
| C42-C43-C47 | 119.2(3) |

| | |
|---|---|
| C44-C43-C47 | 118.5(3) |
| C45-C44-C43 | 117.7(3) |
| C45-C44-H44A | 121.2 |
| C43-C44-H44A | 121.2 |
| C44-C45-C46 | 121.6(3) |
| C44-C45-C48 | 118.9(3) |
| C46-C45-C48 | 119.2(3) |
| C45-C46-C41 | 120.1(3) |
| C45-C46-H46A | 120.0 |
| C41-C46-H46A | 120.0 |
| F7-C47-F8' | 93.8(4) |
| F7-C47-F9 | 104.7(4) |
| F8'-C47-F9 | 119.6(4) |
| F7-C47-F9' | 113.4(4) |
| F8'-C47-F9' | 107.3(4) |
| F9-C47-F9' | 13.7(3) |
| F7-C47-F8 | 107.9(4) |
| F8'-C47-F8 | 15.9(3) |
| F9-C47-F8 | 107.6(4) |
| F9'-C47-F8 | 94.4(4) |
| F7-C47-F7' | 11.9(4) |
| F8'-C47-F7' | 105.7(4) |
| F9-C47-F7' | 98.1(4) |
| F9'-C47-F7' | 108.7(4) |
| F8-C47-F7' | 119.6(4) |
| F7-C47-C43 | 114.7(4) |
| F8'-C47-C43 | 113.0(3) |

| | |
|---|---|
| F9-C47-C43 | 110.0(3) |
| F9'-C47-C43 | 112.9(3) |
| F8-C47-C43 | 111.6(3) |
| F7'-C47-C43 | 109.0(3) |
| F11-C48-F10 | 108.6(4) |
| F11-C48-F12' | 110.2(4) |
| F10-C48-F12' | 91.6(4) |
| F11-C48-F12 | 97.4(4) |
| F10-C48-F12 | 108.0(4) |
| F12'-C48-F12 | 17.9(3) |
| F11-C48-F10' | 100.9(4) |
| F10-C48-F10' | 13.5(4) |
| F12'-C48-F10' | 104.9(4) |
| F12-C48-F10' | 120.7(4) |
| F11-C48-F11' | 8.9(5) |
| F10-C48-F11' | 113.4(4) |
| F12'-C48-F11' | 116.6(4) |
| F12-C48-F11' | 102.3(4) |
| F10'-C48-F11' | 104.2(4) |
| F11-C48-C45 | 117.2(4) |
| F10-C48-C45 | 114.6(4) |
| F12'-C48-C45 | 111.7(3) |
| F12-C48-C45 | 109.5(3) |
| F10'-C48-C45 | 110.7(3) |
| F11'-C48-C45 | 108.4(4) |
| O5-C49-N7 | 125.8(3) |
| O5-C49-H49A | 117.1 |

| | |
|---|---|
| N7-C49-H49A | 117.1 |
| N7-C50-H50A | 109.5 |
| N7-C50-H50B | 109.5 |
| H50A-C50-H50B | 109.5 |
| N7-C50-H50C | 109.5 |
| H50A-C50-H50C | 109.5 |
| H50B-C50-H50C | 109.5 |
| N7-C51-H51A | 109.5 |
| N7-C51-H51B | 109.5 |
| H51A-C51-H51B | 109.5 |
| N7-C51-H51C | 109.5 |
| H51A-C51-H51C | 109.5 |
| H51B-C51-H51C | 109.5 |

Symmetry transformations used to generate equivalent atoms:

**Table S4.** Anisotropic displacement parameters ($Å^2$x $10^3$) for park4.

The anisotropic displacement factor exponent takes the form: $-2\pi^2$[ $h^2$ $a^{*2}U^{11}$ + ... + 2 h k a* b* $U^{12}$ ].

| | $U^{11}$ | $U^{22}$ | $U^{33}$ | $U^{23}$ | $U^{13}$ | $U^{12}$ |
|---|---|---|---|---|---|---|
| Ni1 | 14(1) | 10(1) | 12(1) | 0(1) | 0(1) | 1(1) |
| O1 | 23(1) | 11(1) | 18(1) | 0(1) | -5(1) | 2(1) |
| O2 | 20(1) | 13(1) | 18(1) | -2(1) | -4(1) | 2(1) |
| O3 | 10(1) | 19(1) | 26(1) | 0(1) | 3(1) | 1(1) |
| O4 | 12(1) | 20(1) | 20(1) | 4(1) | 4(1) | 4(1) |
| O5 | 40(1) | 25(1) | 28(1) | 3(1) | 4(1) | 8(1) |
| N1 | 30(1) | 10(1) | 22(1) | 0(1) | -4(1) | 5(1) |
| N2 | 26(1) | 10(1) | 25(1) | 1(1) | -7(1) | 2(1) |
| N3 | 10(1) | 17(1) | 22(1) | -3(1) | 0(1) | 0(1) |
| N4 | 9(1) | 17(1) | 22(1) | -3(1) | 4(1) | 2(1) |
| N5 | 11(1) | 19(1) | 16(1) | 4(1) | 2(1) | 3(1) |
| N6 | 11(1) | 18(1) | 22(1) | 5(1) | 5(1) | 3(1) |
| N7 | 33(2) | 20(1) | 19(1) | 0(1) | 4(1) | 4(1) |
| C2 | 20(2) | 11(2) | 34(2) | -4(1) | -3(1) | 1(1) |
| C3 | 42(2) | 14(2) | 27(2) | 2(1) | 6(2) | 8(1) |
| C5 | 21(2) | 15(2) | 31(2) | 5(1) | -5(1) | 2(1) |
| C7 | 34(2) | 12(2) | 20(2) | -4(1) | -7(1) | 1(1) |
| C8 | 17(1) | 14(2) | 18(2) | 2(1) | 0(1) | 0(1) |
| C9 | 12(1) | 14(2) | 16(1) | 1(1) | 2(1) | 0(1) |
| C10 | 14(1) | 12(1) | 19(1) | 1(1) | 2(1) | 2(1) |
| C11 | 15(1) | 14(2) | 17(1) | 2(1) | 1(1) | 2(1) |
| C12 | 12(1) | 19(2) | 16(1) | -1(1) | 1(1) | 0(1) |

| | | | | | | |
|---|---|---|---|---|---|---|
| C13 | 17(1) | 12(2) | 20(2) | -3(1) | 2(1) | -2(1) |
| C14 | 14(1) | 13(2) | 21(2) | -1(1) | 0(1) | 1(1) |
| C15 | 15(1) | 16(2) | 24(2) | 0(1) | 2(1) | -1(1) |
| C16 | 26(2) | 18(2) | 28(2) | -1(1) | 7(1) | 2(1) |
| C17 | 22(2) | 14(2) | 29(2) | 4(1) | -1(1) | 0(1) |
| C18 | 23(2) | 15(2) | 20(2) | 3(1) | -4(1) | 3(1) |
| C19 | 16(1) | 12(1) | 13(1) | 1(1) | 3(1) | 0(1) |
| C20 | 15(1) | 14(2) | 14(1) | 2(1) | 3(1) | 1(1) |
| C21 | 14(1) | 12(1) | 14(1) | 2(1) | 3(1) | 1(1) |
| C22 | 17(1) | 13(1) | 15(1) | -1(1) | 3(1) | -1(1) |
| C23 | 14(1) | 18(2) | 10(1) | 2(1) | 2(1) | -1(1) |
| C24 | 14(1) | 16(2) | 14(1) | 3(1) | 4(1) | 2(1) |
| C25 | 18(1) | 13(2) | 20(2) | -1(1) | 0(1) | 1(1) |
| C26 | 16(1) | 22(2) | 40(2) | -8(2) | 1(1) | 2(1) |
| C27 | 39(2) | 14(2) | 20(2) | 0(1) | -6(1) | 4(1) |
| C28 | 20(1) | 15(2) | 24(2) | -4(1) | 0(1) | 3(1) |
| C29 | 16(1) | 17(2) | 18(2) | 1(1) | -4(1) | 0(1) |
| C30 | 13(1) | 17(2) | 12(1) | 2(1) | -1(1) | 1(1) |
| C31 | 14(1) | 14(2) | 13(1) | 1(1) | 1(1) | 0(1) |
| C32 | 11(1) | 18(2) | 18(1) | 1(1) | 2(1) | 2(1) |
| C33 | 13(1) | 18(2) | 16(1) | 0(1) | 1(1) | 0(1) |
| C34 | 17(1) | 16(2) | 17(1) | 0(1) | 1(1) | 2(1) |
| C35 | 16(1) | 18(2) | 15(1) | 2(1) | 2(1) | 1(1) |
| C36 | 12(1) | 18(2) | 16(1) | 0(1) | 1(1) | 0(1) |
| C37 | 16(1) | 17(2) | 29(2) | -4(1) | 1(1) | 1(1) |
| C38 | 18(1) | 19(2) | 28(2) | -3(1) | -2(1) | 2(1) |
| C39 | 14(1) | 16(2) | 17(1) | 1(1) | 1(1) | 0(1) |

| | | | | | | |
|---|---|---|---|---|---|---|
| C40 | 12(1) | 16(2) | 13(1) | -1(1) | 0(1) | 0(1) |
| C41 | 15(1) | 14(2) | 16(1) | -2(1) | 2(1) | 1(1) |
| C42 | 15(1) | 15(2) | 17(1) | 0(1) | 2(1) | 3(1) |
| C43 | 18(1) | 17(2) | 18(2) | -2(1) | 3(1) | 1(1) |
| C44 | 26(2) | 16(2) | 16(1) | 1(1) | 3(1) | 4(1) |
| C45 | 23(2) | 16(2) | 15(1) | -4(1) | 0(1) | 4(1) |
| C46 | 16(1) | 16(2) | 18(1) | -1(1) | 2(1) | 1(1) |
| C47 | 24(2) | 19(2) | 23(2) | 3(1) | 10(1) | 2(1) |
| C48 | 27(2) | 21(2) | 18(2) | 0(1) | -2(1) | 5(1) |
| C49 | 32(2) | 21(2) | 19(2) | -3(1) | 6(1) | -1(1) |
| C50 | 42(2) | 28(2) | 39(2) | 1(2) | 9(2) | -3(2) |
| C51 | 45(2) | 33(2) | 26(2) | 1(2) | 2(2) | 11(2) |

**Table S5.** Hydrogen coordinates ( × $10^4$) and isotropic displacement parameters (Å$^2$ × $10^3$) for park4.

| | x | y | z | U(eq) |
|---|---|---|---|---|
| H3 | 9010(40) | -10(30) | 2759(19) | 35(11) |
| H4 | 9730(40) | 1550(30) | 3143(16) | 17(9) |
| H5 | -4600(40) | -1560(20) | -2851(16) | 17(8) |
| H6 | -4780(40) | -390(30) | -3556(17) | 27(10) |
| H1A | 3539 | 1477 | -316 | 16 |
| H6A | 711 | 1013 | 68 | 18 |
| H1'A | 1408 | 1146 | 389 | 19 |
| H6'A | 2865 | 1347 | -763 | 15 |
| H2A | 4071 | 2564 | 545 | 26 |
| H2B | 2716 | 2127 | 888 | 26 |
| H2C | 4035 | 2399 | 345 | 26 |
| H2D | 3092 | 2332 | 945 | 26 |
| H3A | 1998 | 3605 | 547 | 33 |
| H3B | 2807 | 3582 | -89 | 33 |
| H3C | 2815 | 3808 | 233 | 33 |
| H3D | 1374 | 3238 | 438 | 33 |
| H4A | 577 | 3538 | -467 | 25 |
| H4B | 91 | 2755 | 69 | 25 |
| H4'A | 780 | 3532 | -658 | 22 |
| H4'B | 2301 | 3215 | -841 | 22 |
| H5A | -102 | 1788 | -978 | 27 |
| H5B | 1562 | 2139 | -1052 | 27 |

| | | | | |
|---|---|---|---|---|
| H5C | 538 | 1912 | -1212 | 27 |
| H5D | 19 | 1832 | -498 | 27 |
| H7A | 4608 | 1066 | 1035 | 28 |
| H11A | 5971 | -2603 | 2227 | 18 |
| H13A | 6106 | 422 | 1895 | 20 |
| H15A | 1992 | -2988 | 879 | 28 |
| H15B | 2120 | -3262 | 1640 | 28 |
| H15C | 1961 | -4160 | 1090 | 28 |
| H16A | 4104 | -3344 | 236 | 36 |
| H16B | 3973 | -4495 | 485 | 36 |
| H16C | 5490 | -3817 | 616 | 36 |
| H17A | 4362 | -3933 | 2198 | 33 |
| H17B | 5640 | -4175 | 1784 | 33 |
| H17C | 4120 | -4849 | 1659 | 33 |
| H18A | -112 | 366 | -1323 | 24 |
| H22A | -996 | -3745 | -2176 | 18 |
| H24A | -1754 | -783 | -1987 | 17 |
| H26A | 3101 | -3244 | -1921 | 39 |
| H26B | 3690 | -4042 | -1392 | 39 |
| H26C | 3411 | -2910 | -1159 | 39 |
| H27A | 431 | -4464 | -631 | 38 |
| H27B | 1769 | -3660 | -367 | 38 |
| H27C | 2049 | -4791 | -600 | 38 |
| H28A | 44 | -5040 | -1819 | 30 |
| H28B | 1689 | -5319 | -1760 | 30 |
| H28C | 1091 | -4563 | -2315 | 30 |
| H29A | 6662 | -872 | 3155 | 21 |

| | | | | |
|---|---|---|---|---|
| H29B | 7851 | -1467 | 2846 | 21 |
| H32A | 11015 | 2894 | 3723 | 18 |
| H34A | 8969 | 5430 | 4067 | 20 |
| H36A | 6680 | 2884 | 3183 | 18 |
| H39A | -2823 | -2978 | -2961 | 19 |
| H39B | -3724 | -2927 | -2352 | 19 |
| H42A | -1306 | 212 | -3985 | 19 |
| H44A | -2611 | 2405 | -5253 | 23 |
| H46A | -5553 | 771 | -4302 | 20 |
| H49A | 2603 | 8848 | 2559 | 29 |
| H50A | -483 | 7396 | 2775 | 54 |
| H50B | 251 | 6991 | 3459 | 54 |
| H50C | 285 | 6347 | 2791 | 54 |
| H51A | 3844 | 7627 | 3087 | 51 |
| H51B | 3048 | 6496 | 2985 | 51 |
| H51C | 3009 | 7132 | 3655 | 51 |

**Table S6.** Hydrogen bonds for park4 [Å and °].

| D-H...A | d(D-H) | d(H...A) | d(D...A) | <(DHA) |
|---|---|---|---|---|
| N3-H3...O5#1 | 0.79(4) | 2.28(4) | 2.983(3) | 149(4) |
| N4-H4...O4#2 | 0.82(3) | 2.04(3) | 2.837(3) | 162(3) |
| N5-H5...O3#3 | 0.89(3) | 2.08(3) | 2.906(3) | 154(3) |
| N6-H6...O3#3 | 0.86(4) | 2.06(4) | 2.849(3) | 153(3) |

Symmetry transformations used to generate equivalent atoms:

#1 x+1,y-1,z #2 -x+1,-y,-z #3 -x,-y,-z

**6. Mass spectrum of a bis-urea salen aluminum catalyst (3b)**

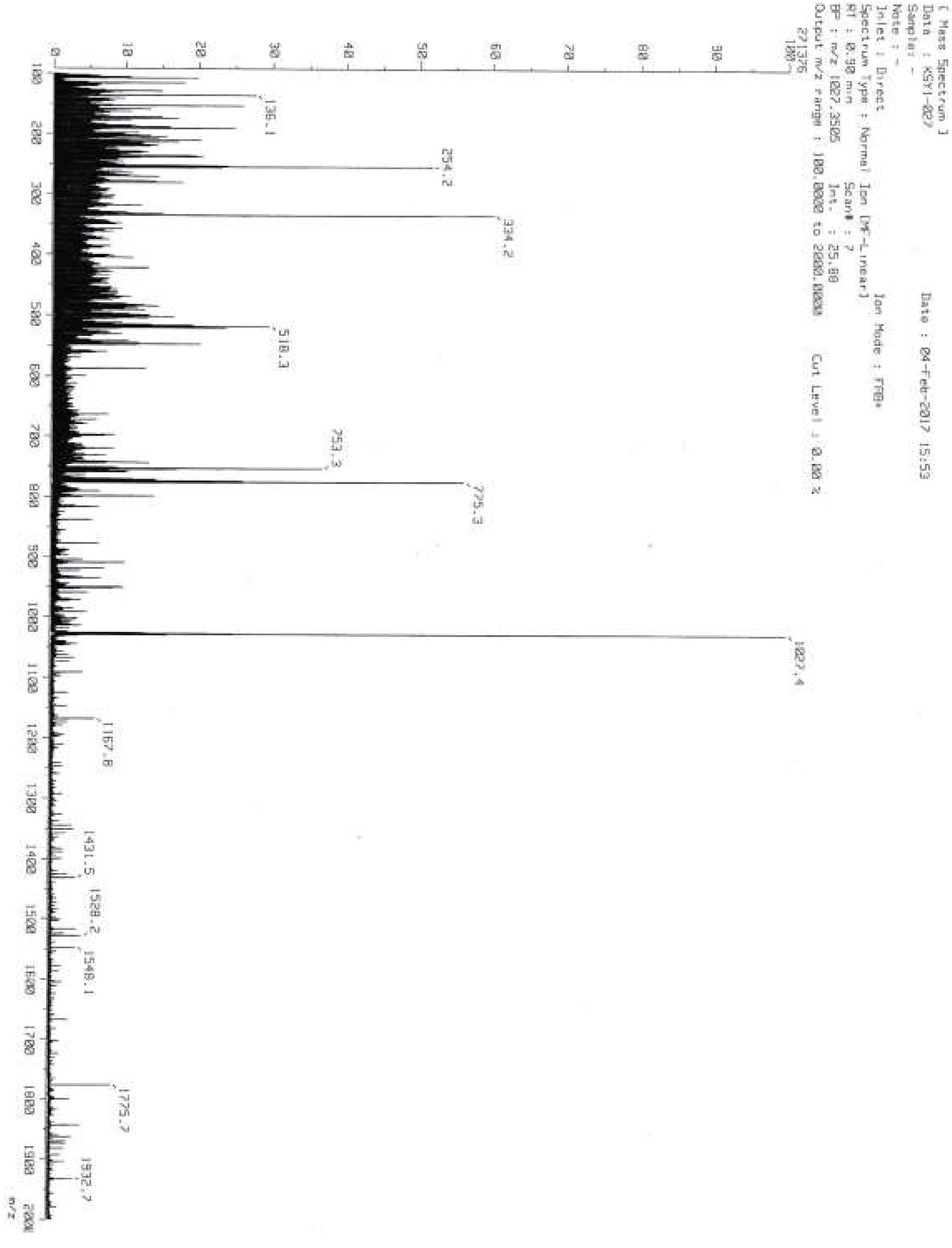

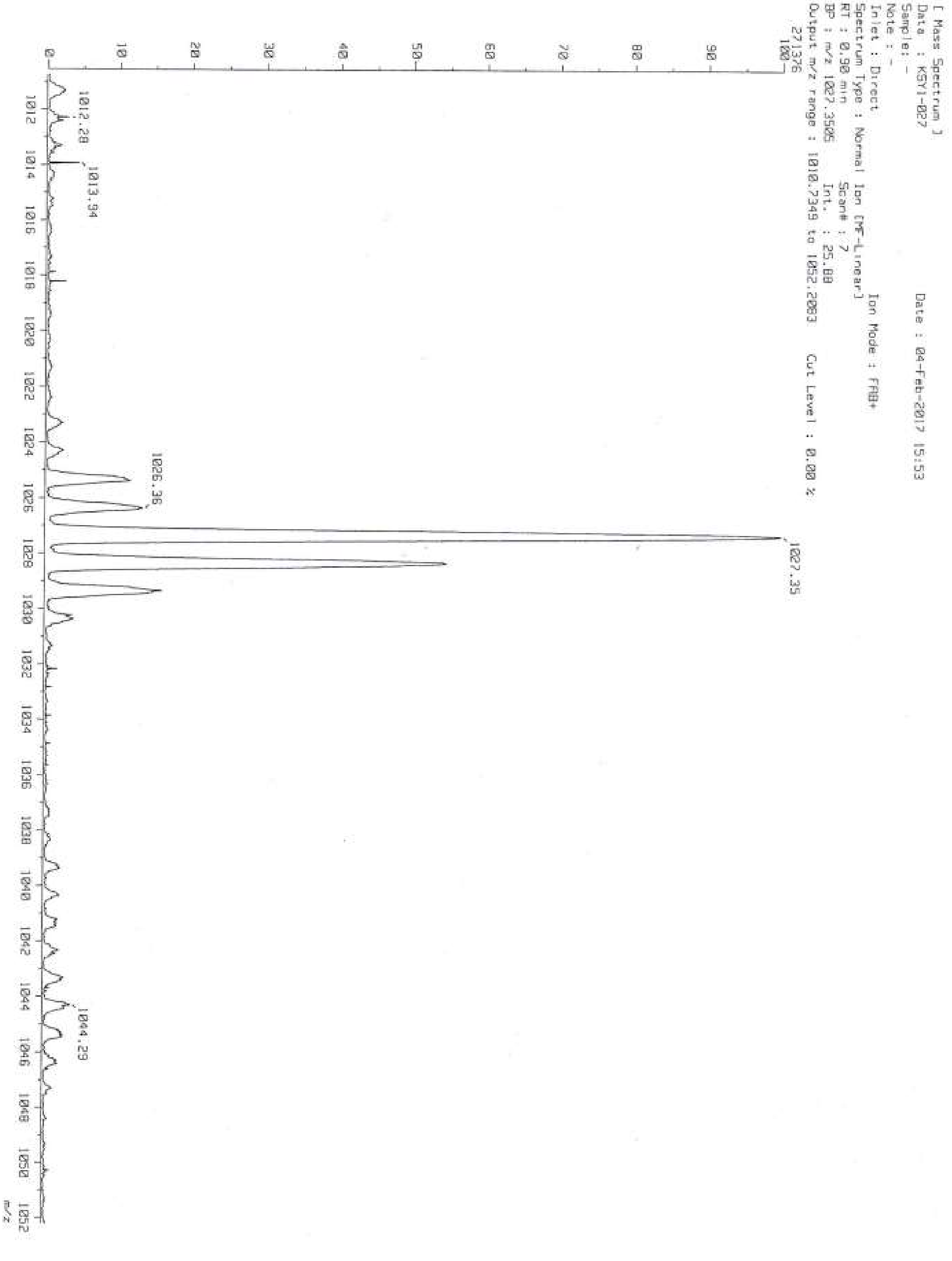
[ Mass Spectrum ]
Data : KSY1-027
Date : 04-Feb-2017 15:53
Sample: -
Note : -
Inlet : Direct
Ion Mode : FAB+
Spectrum Type : Normal Ion [MF-Linear]
RT : 0.90 min
Scan# : 7
BP : m/z 1027.3505
Int. : 25.88
Output m/z range : 1010.7349 to 1052.2083
Cut Level : 0.00 %
271376
1027.35
1026.36
1012.28
1013.94
1044.29
m/z

[ Mass Spectrum ]

Page: 1

Data : KSY1-027 Date : 04-Feb-2017 15:53
Sample: -
Note : -
Inlet : Direct Ion Mode : FAB+
Spectrum Type : Normal Ion [MF-Linear]
RT : 0.90 min Scan# : 7
BP : m/z 1027.3505 Int. : 25.88
Output m/z range : 1011.0000 to 1052.0000 Cut Level : 1.00 %

| m/z | Int. | Norm. |
|---|---|---|
| 1011.3302 | 0.61 | 2.36 |
| 1012.2801 | 0.76 | 2.92 |
| 1012.4105 | 0.55 | 2.11 |
| 1013.3049 | 0.50 | 1.94 |
| 1013.3981 | 0.29 | 1.12 |
| 1013.9387 | 1.11 | 4.30 |
| 1017.8764 | 0.31 | 1.19 |
| 1018.2126 | 0.68 | 2.64 |
| 1023.3008 | 0.61 | 2.34 |
| 1024.2938 | 0.61 | 2.36 |
| 1024.5187 | 0.27 | 1.05 |
| 1025.3809 | 3.03 | 11.69 |
| 1026.3561 | 3.45 | 13.32 |
| 1027.3505 | 25.88 | 100.00 |
| 1028.3453 | 14.15 | 54.68 |
| 1029.3407 | 4.15 | 16.02 |
| 1030.3553 | 1.02 | 3.94 |
| 1031.3329 | 0.29 | 1.11 |
| 1032.1604 | 0.46 | 1.79 |
| 1032.8190 | 0.26 | 1.00 |
| 1033.8541 | 0.26 | 1.00 |
| 1039.3771 | 0.58 | 2.25 |
| 1040.3774 | 0.54 | 2.10 |
| 1041.2084 | 0.51 | 1.99 |
| 1042.3230 | 0.55 | 2.13 |
| 1042.4930 | 0.32 | 1.22 |
| 1043.3059 | 0.73 | 2.82 |
| 1044.2893 | 0.96 | 3.72 |
| 1045.3677 | 0.72 | 2.78 |
| 1046.4088 | 0.52 | 2.01 |
| 1047.3179 | 0.34 | 1.30 |

7. **FTIR spectrum of bis-urea salen aluminum catalyst (3b)**

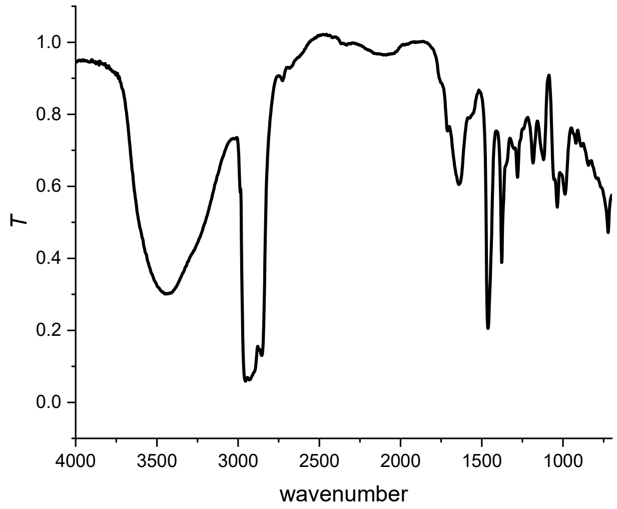


FTIR spectrum of bis-urea salen aluminum catalyst (**3b**) powder.

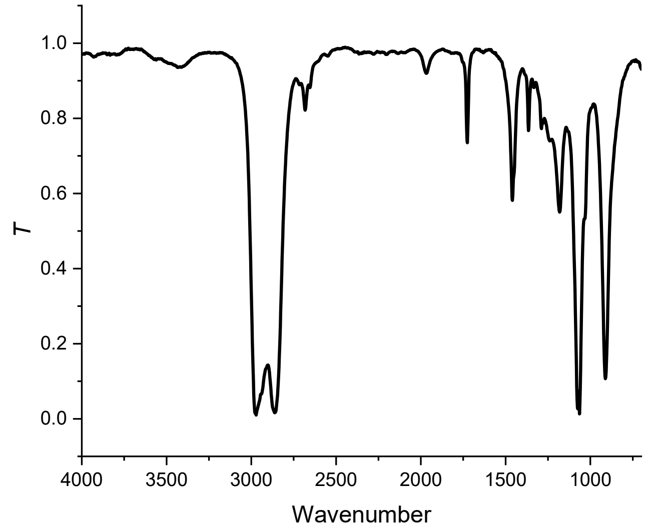


FTIR spectrum of bis-urea salen aluminum catalyst (**3b**) in THF solution (3 mM).

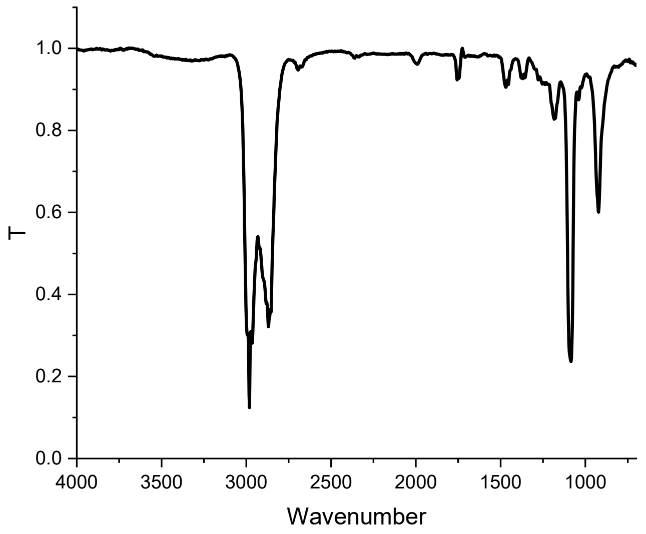


FTIR spectrum of bis-urea salen aluminum catalyst (**3b**) in THF solution (1.5 mM).

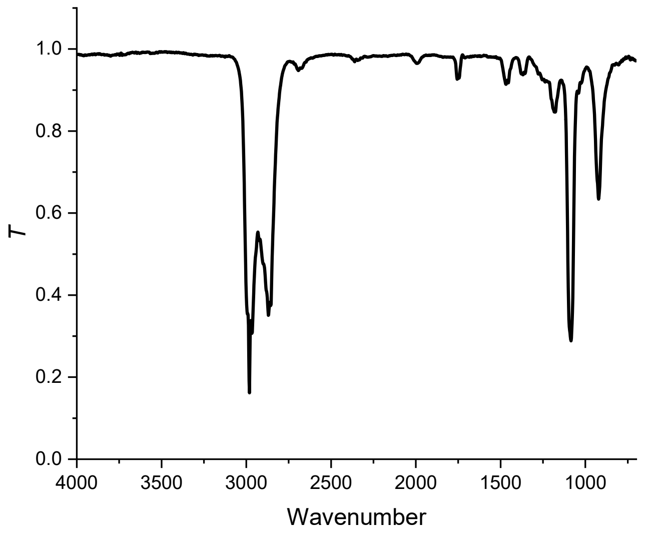


FTIR spectrum of bis-urea salen aluminum catalyst (**3b**) in THF solution (1 mM).
